# The Health of the Collier

**Published:** 1919-05-10

**Authors:** 


### THE HEALTH OF THE COLLIER.

### His Comparative Immunity from Phthisis.

The chief cause of disablement in coal-mines is most probably simple injury. Explosions, which kill and maim their thousands, do not happen often; although, as they get into the papers, their renown is great. But injury claims its tens of thousands. There are many chances of trauma down amongst moving wire-ropes, moving tubs, restive ponies, and falls of stone. These last often crush a man like a beetle, an occurrence of which the outside public never hears. Working men run far more risk of nasty injury than the middle class, and the collier's job is one of the riskiest of all.

As for sickness, nystagmus—an ocular affection the exact causation of which is disputed, but which is undoubtedly an occupational disease—and in wet mines rheumatism, are perhaps the commonest disorders. General debility is not infrequent. When discussing the health of coal-miners, especially old ones, it should not be forgotten that a good few leave the work, at all events underground employment, because their health will not stand the strain of it. Washing restores a collier's looks by taking off the smut, but nothing will give him a ruddy colour. The fish-belly skin complexion is almost universal. Lung disease is much less common nowadays, doubtless because of the improvements to ventilation. Miners' phthisis, first described by Ramazzini in the eighteenth century, and which is a scarring of the lungs from local irritation, upon which tuberculosis develops, affects the gold-miner and the tin-miner, but rarely the coal-miner. An early theory to account for this incidence was simple and reasonable sounding enough, being to the effect that the silicious flinty dust inhaled in Cornwall and on the Rand was of a hard, cutting consistency, likely to damage the lung mechanically, whereas coal-dust was soft. But Poincaré's *dictum* that each generation laughs at the generalisations of the preceding one was soon, oversoon, fulfilled, for discrepancies cropped up. In India and elsewhere there were gold-mines in silicious rock, with no silicosis amongst the workers. Another bit of disproof came to notice in a curious way. Practical engineers observed that in colliery explosions, notably one at Altofts in Yorkshire, the path of the explosion avoided tracks where stone dust was mixed with the coal dust; the stone seemed to deaden the coal and prevent it igniting. The plain indication to put more stone dust into the mines was followed, and it is now generally agreed that this measure has much lessened the risk of explosion. Now the stone dust used was shale; and amongst well-informed persons there was a good deal of puzzlement when, in 1913, Dr. Mellor found in this shale a considerable percentage of crystalline silica, just the same substance that is so deleterious in the Transvaal. Yet happily the colliers did not suffer from inhaling what had been strewed about to protect them from explosions. Authorities now think that the matter turns upon whether the silica-dust is pure or not. Unadulterated silica dust, like that ground by rock drills from the pure Transvaal quartz, is deadly: but at the Indian gold-mines aforementioned the rock of the country is not nearly so pure. There is an admixture of other dust, which, so Dr. Haldane's experiments would suggest, the epithelial cells lining the air-cavities of the lungs can seize on and eliminate. More than this, these removable dust particles seem to act on silicious dust in a physical way (adsorption, or solid solution, is the technical term) so as to make possible their arrest too. But without this false friend, powdered silica defies the local pulmonary authorities only too successfully. A hint of this theory had been given in 1915 by Dr. H. C. Ross, who found that the presence of clay in firebrick-making modified greatly the pathogenicity of the silica dust. This hypothesis will also obviously account for the innocuousness of silicious stone-dust put down the mines among the black coal-dust as a safety measure. Indeed, when a South African mining engineer heard of it, he made the good suggestion to try to get Rand gold-miners work in coal or other harmless dust, week about with their ordinary occupation.

But when Dr. Haldane claims that the comparative—only comparative—freedom of coal-miners from ordinary consumption is due to their inhalation of coal-dust, which does good by acting as decoy to the tubercle bacilli, as it may do to the tiny pellets of silica; when he jumps from inorganic dust particles to living germs, then he is guessing. Where is the proof that particles of coal can "absorb" the bacillus of Koch? And *a fortiori* the coal-owner is wrong to speak of his dusty pit, on the strength of Dr. Haldane's excellent work, as an anti-tuberculosis agency. What tuberculosis officer, or what sanatorium superintendent, in a mining area lets a recovered consumptive collier go down the pit again, if he can help it? Relapse would be the only too probable result. It may be granted that colliery surgeons find contamination with coal-dust no impediment at all to the speedy healing of wounds. But quite apart from any specific action of coal-dust there need be no mystery about the comparatively low tuberculosis mortality-rate of colliers. A coal-mine is a very big place, running for miles underground, and work there is devoid of the stuffiness of the factory, approximating to open-air conditions now that better ventilation is provided. The danger of the job excludes low-grade remuneration. These two factors will tend to reduce tuberculosis anywhere. And on top of this, it chances that coal-dust in moderate quantities can be breathed with impunity by the ordinary man. If it could not, the history of the world these last two centuries, and very particularly the history of England, would have been utterly different.

